# A convenient UHPLC-MS/MS method for routine monitoring of plasma and brain levels of nicotine and cotinine as a tool to validate newly developed preclinical smoking model in mouse

**DOI:** 10.1186/s12868-017-0389-5

**Published:** 2017-10-11

**Authors:** Mohammad A. Kaisar, Raja Reddy Kallem, Ravi K. Sajja, Ali Ehsan Sifat, Luca Cucullo

**Affiliations:** 1 Department of Pharmaceutical Sciences, School of Pharmacy, Texas Tech University Health Sciences Center, 1300 S. Coulter Street, Amarillo, TX 79106 USA; 2 Office of Sciences, School of Pharmacy, Texas Tech University Health Sciences Center, Amarillo, TX 79106 USA; 3 Center for Blood Brain Barrier Research, Texas Tech University Health Sciences Center, Amarillo, TX 79106 USA

**Keywords:** Nicotine, Cotinine, Mouse plasma, Preclinical, animal model, Tobacco smoke, Method validation, UHPLC-MS/MS

## Abstract

**Background:**

A sensitive, rapid and selective UHPLC–MS/MS method has been developed and validated for the quantification of Nicotine (NT) and Cotinine (CN) using Continine-*d*_3_ as internal standard (IS) as per FDA guidelines. Sample preparation involved simple protein precipitation of 20 µL mouse plasma or brain homogenate using acetonitrile at 1:8 ratio. Mass Spectrometer was operated in positive polarity under the multiple reaction-monitoring mode using electro spray ionization technique and the transitions of *m/z* 163.2 → 132.1, 177.2 → 98.0 and 180.2 → 101.2 were used to measure the NT, CN and IS, respectively. The elution of NT, CN and IS are at 1.89, 1.77 and 1.76 min, respectively. This was achieved with a gradient mobile phase consisting of 5 mM ammonium bicarbonate, acetonitrile and methanol (3:1, v/v) at a flow rate of 0.3 mL/min on a Kinetex EVO C18 column. The method was validated with a lower limit of quantitation 3.0 ng/mL in mouse plasma and brain for both the analytes.

**Results:**

A linear response function was established for the range of concentrations 3–200 (*r* > 0.995) for NT and 3–600 ng/mL (*r* > 0.995) for CN. The intra- and inter-day precision values met the acceptance criteria. NT and CN are stable in the battery of stability studies viz., stock solution, bench-top and auto-sampler.

**Conclusion:**

This method was successfully utilized to validate a newly developed preclinical smoking model in mice.

## Background

Tobacco smoking (TS) adversely impacts public health by contributing to enormous health-related economic losses and countless deaths each year. Exposure to tobacco constituents through active or passive smoking harms several organs resulting in an increased risk of lung diseases, cardio/cerebrovascular disorders and cancers [1]. TS is considered as the leading cause of preventable disease and death in the United States, accounting for more than 480,000 deaths every year, or 1 of every 5 deaths [2]. In 2015, about 15 of every 100 U.S. adults aged 18 years or older smoked cigarettes. This means an estimated 36.5 million adults in the United States currently smoke cigarettes. More than 16 million Americans live with a smoking-related disease.

Nicotine (NT) is the major bioactive and addictive constituent of TS and is rapidly metabolized into cotinine (CN) and other less abundant metabolites primarily by the liver cytochrome P450s. The plasma level of NT in chronic smokers generally average between 20 and 60 ng/mL although depending on a number of variables (including volume, depth of inhalation, and frequency; type of cigarette smoked and relative nicotine yield; number, age, and gender of the sample population being tested as well as the time the samples were collected during testing) plasma nicotine concentration can raise up to 100 ng/mL [3, 4]. By contrast, the plasma concentration of CN is significantly higher (ranging between 250 and 350 ng/mL) and remains fairly stable. NT has a very short half-life of ~ 2 h but its primary metabolite CN has significantly higher plasma half-life of approximately 16 h [3]. Because of the longer half-life CN is considered a well established biomarker for TS exposure [3, 5–7]. Investigational studies to comprehend the mechanisms underlying the CNS effects of NT in biological tissues do require sensitive and specific measurement of both the parent compound and its metabolite CN originated from TS.

Preclinical studies using animal models with unique protocols are critically important to identify biomarkers for TS related disorders, dissect their pathophysiology and to assess the preventive/therapeutic efficacy of drugs prior to any clinical trial. If the target organ of interest is brain, working on clinical subjects is nearly impossible since it is an invasive terminal procedure. Several animal models and different protocols have been used for preclinical research. This includes, transdermal implantation of osmotic mini pump [6, 8], IV injection of nicotine/tobacco extract solution [9, 10], direct inhalation of TS in animal restraint device [11]. Most of these exposure conditions fail to simulate realistic smoking behavior. Concerning the appropriate rodent model to use to study the impact of smoking in humans the animal of choice is primarily dependent upon the objective of the studies. In fact, while mice seem better-suited models to simulate the nicotine metabolism in humans based on structural and functional similarities of the respective metabolizing enzymes (in humans nicotine is primarily metabolize by the cytochrome P450 enzyme CYP2A6 which shares close functional efficiency [12, 13] and 84% amino acid sequence similarity [14, 15] with that of the mice; the CYP2A5); rats (where nicotine metabolism is instead largely under the control of P450 CYP2B family of enzymes [16]) seems to provide a more translationally relevant pharmacokinetic model when considering the effective rate of nicotine elimination (the nicotine T1/2 in humans is approximately 2 h while in rats is about 1 h and only 15 min in mice [17–19]). Despite these differences, both rats and mice serve as purposeful and translatable models for preclinical smoking research. Herein, we report a newly developed preclinical smoking model using C57BL/6 mice. Our exposure condition mimics the behavior, intensity and smoking pattern of a chronic heavy smokers and can be used either for acute or long term in vivo studies.

Abundant literature available on simultaneous estimation of NT and CN in human plasma, serum, urine and saliva using gas chromatography-mass spectrometry (GC-MS) and liquid chromatography-tandem mass spectrometry (LC–MS/MS) technics. Recent publication by Abdallah et al. [20] and Tretzel et al. [21] have comprehensively discussed on the various bioanalytical methods published for the simultaneous quantification of NT and CN in human serum, plasma or urine samples. Whereas Vieira-Brock et al. [22] have extensively discussed about all currently available methods for the estimation of NT and CN in rat plasma or brain homogenate. All these groups opined that most of these reported methods (GC, CE and LC–MS/MS) had the limitation of sensitivity, tedious sample preparation procedure, very long run times and/or higher sample volumes. Though Alsharari et al. [23] mentioned some details of their method in mouse plasma, the main limitations of the method were 200 µL sample volume, longer procedure for sample preparation and poor sensitivity. Therefore, we required to develop and validate a sensitive, rapid, cost effective and simple UHPLC-MS/MS method for the simultaneous estimation of NT and CN in mice plasma and brain samples. This newly developed method was successfully utilized to validate our preclinical smoking model in mice demonstrating the extent of exposure to TS.

## Methods

### Chemicals and reagents

**Fig. 1:**
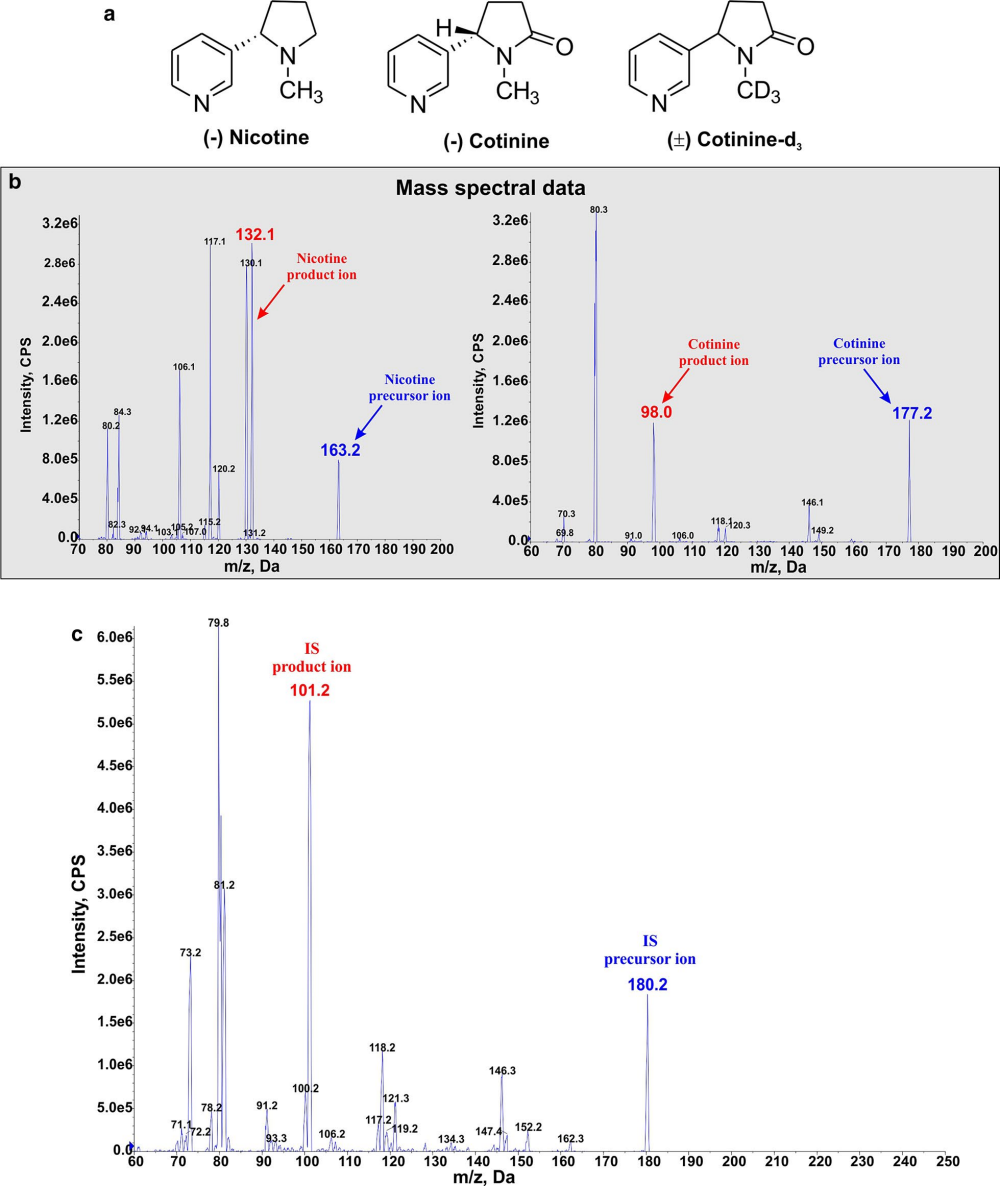
Mass spectral data and representative peaks. Chemical structure of analytes and IS (**a**), mass spectral data of nicotine and cotinine (**b**). **c** Mass spectral data for the Internal Standards

(−)-Nicotine (Cat # 36733); (−)-Cotinine, 1 mg/mL solution in methanol (Cat # C0430); deuterated internal standard (IS) (±)-Cotinine-*d*_3_ (see Fig. 1a), 1 mg/mL solution in methanol (Cat # C-035) and LC/MS grade eluent additive ammonium bicarbonate (Cat # 40867) were purchased from Sigma-Aldrich, USA. LC/MS grade solvents water (Cat # W6-4), acetonitrile (Cat # A955-4) and methanol (Cat # A456-4) were obtained from Fisher Scientific, USA. Tobacco reference product, 3R4F equivalent to regular cigarettes were purchased from University of Kentucky.

### UHPLC-MS/MS instrumentation

The UHPLC–MS/MS system equipped with AB SCIEX QTRAP® 5500 mass spectrometer (Foster City, CA, USA) attached to a Nexera UHPLC system from Shimadzu Corporation (Columbia, MD) was used. The liquid chromatographic unit consisted of a Sil-30AC auto-sampler, LC-30AD binary pumps, a CBM-20A controller, a DGA-20A5 degasser, and a CTO-30A column oven. Data acquisition and quantitation were performed by Analyst software.

### Chromatographic conditions

Kinetex EVO C18 column (100 × 2.1 mm I.D., 1.7 µm particle size, 100 Å pore size) proceeded by SecurityGuard™ ULTRA guard column (Phenomenex; Torrance, CA, USA) was used to perform the chromatographic separation while column oven was set at 40 °C temperature. The injection volume was 4 µL. For the elution of the analytes, gradient flow of a mixture of 5 mM ammonium bicarbonate buffer (pH not adjusted) (A) and Acetonitrile: Methanol (3:1, v/v) (B) has been used at a flow rate of 0.3 mL/min. The gradient program started with 10% B, kept unaltered for 0.40 min which was then raised to 90% B over 0.8 min. From 0.8 to 2.2 min the mobile phase composition remained constant followed by switching back to the initial condition in next 0.2 min. The column was re-equilibrated at 10% B for another 0.6 min. Total run time of the method is 3.0 min with retention time (RT) for both analytes and IS were 1.89 (nicotine), 1.77 (cotinine) and 1.76 (cotinine-*d*_*3*_) minute, respectively.

### Mass spectrometric conditions

A triple quadrupole mass spectrometer (5500 QTRAP; Foster City, CA, USA) was used for the quantitation of NT, CN and IS. The ionization source was through electro-spray ionization (TurbolonSpray^®^), and the analytes were detected using multiple reactions monitoring (MRM) operated in the positive mode by monitoring their transition pairs of *m/z* 163.2 precursor ion to the *m/z* 132.1 for NT, *m/z* 177.2 precursor ion to the *m/z* 98.0 for CN, and *m/z* 180.2 precursor ion to the *m/z* 101.2 product ion for the I.S (see Fig. 1b). The corresponding mass spectral data for the Internal Standards are also shown in Fig. 1c.

Quadrupoles Q1 and Q3 were set on unit resolution with the help of Analyst software™ (version 1.6.2). The source/gas and compound parameters were optimized to obtain the highest [M−H]+ ion abundance by infusing the standard solutions of the analyte of interest via a syringe pump into the mass spectrometer. The optimized source/gas parameters were as follows: curtain gas, 35 psi; collision gas, high; ion spray voltage, 5500 V; temperature, 550 °C; ion source gas 1 (nebulizer gas), 50 psi; and ion source gas 2 (turbo gas), 50 psi. The compound parameters were optimized for each of the analytes and are represented in Table 1. The product ion of NT (*m/z* 132.1), CN (*m/z* 98.0) and IS (*m/z* 101.2) selected in our study for monitoring are similar to previously reported ions [24].

**Table 1:** Compound parameters for analytes and internal standard with MRM in positive electrospray mode

	Q1 mass (Da)	Q3 mass (Da)	DP	CE	CXP	EP
Analytes						
Nicotine	163.2	132.1	100	27	10	10
Cotinine	177.2	98	100	34	10	10
Internal standard						
Cotinine-D3	180.2	101.2	100	31	13	10

*DP* declustering potential, *CE* collision energy, *CXP* collision exit potential, *EP* entrance potential

### Preparation of stock solutions, calibration standards and quality control samples

Stock solution of nicotine: cotinine (1:1, 1 mg/mL each), nicotine: cotinine (1:3, 1: 3 mg/mL) and IS (1 µg/mL) were prepared in methanol and stored in − 20 °C. For plasma, nicotine stock solution was diluted to 30, 50, 100, 200, 300, 400, 500, 1000, 2000 ng/mL (calibration standard) and 30, 90, 750 and 1500 ng/mL (quality control, *QC* samples) in methanol while cotinine stock solution was diluted to 30, 150, 300, 600, 900, 1200, 1500, 3000, 6000 ng/mL (calibration standard) and 30, 90, 2250 and 4500 ng/mL (QC samples) in methanol and stored at − 20 °C. For brain, both the nicotine and cotinine stock solution were diluted to 30, 50, 100, 200, 300, 500, 750, 1000 ng/mL (calibration standard) and 30, 90, 400, 800 ng/mL (QC samples) in methanol. Nicotine: cotinine (1:1) stock solution was used for brain matrix, 30, 90 ng/mL stock for plasma matrix and the remaining dilutions for plasma matrix were made from 1:3 nicotine: cotinine stock. 5 µL (both calibration standard and QC samples) of stock solution was spiked into 45 µL of matrix (either plasma or brain matrix) and vortexed for 30 s. Ten fold dilution of the stock solution by spiking in matrix produced a concentration series of 3, 5, 10, 20, 30, 40, 50, 100, 200 ng/mL (calibration standard) and 3, 9, 75, 150 ng/mL (QC samples) of nicotine; 3, 15, 30, 30, 90.120, 150, 300, 600 ng/mL (calibration standard) and 3, 9, 225, 450 ng/mL (QC samples) of cotinine in plasma; 3, 5, 10, 20, 30, 50, 75, 100 ng/mL (calibration standard) and 3, 9, 40 and 80 ng/mL of both nicotine and cotinine in brain matrix. A linear calibration curve was constructed by plotting ratio of peak area of analyte and IS versus drug concentration. Blank plasma and brain homogenate were collected from control C57BL/6 mice.

### Sample preparation

50-fold dilution of IS stock in acetonitrile provided a concentration of 20 ng/mL. 160 µL of IS solution was added to 20 µL (1:8) of calibration standard, QC sample or test samples (plasma/brain samples collected from mice exposed to smoke) vortexed for 1 min at high speed and centrifuged at 14,000 rpm for 25 min at 4 °C. The remaining 30 µL of calibration standard/QC samples were stored at − 80 °C for future use. The supernatant was transferred carefully into an autosampler inserts for LC-MS/MS analysis. A 4 µL sample was injected onto an analytical column.

### Method validation

Intra and inter-run accuracy and precision of quality control samples at lower limit of quantification (LLOQ), low (LQC), middle (MQC) and high (HQC) quality control concentrations (3, 9, 75, 150 ng/mL of nicotine and 3, 9, 225, 450 ng/mL cotinine in plasma; 3, 9, 40, 80 ng/mL of both nicotine and cotinine in brain) were analyzed against the calibration curve. The accuracy of five repeats of each concentration was estimated using the following equation:

$$Accuracy = \frac{Measured\,concentration}{Nominal\,concentration} \times 100\%$$

Precision was measured as percentage relative standard deviation (R.S.D) of accuracy by the following equation:

$$Precision = \frac{Standard\,deviation\, of\,accuracy}{Mean\,of\,accuracy} \times 100\%$$

The acceptable limits of accuracy were set to 80–120% for LLOQ and 85–115% for LQC, MQC and HQC. Similarly, Precision ≤ 20 for LLOQ and ≤ 15 for low, middle and high concentration were considered acceptable.

### Selectivity

Selectivity of the method was assessed by analyzing six different lots of mouse blank plasma and brain samples. The background noises or interferences responses at the retention time of the NT and CN should be less than 20% of the mean response of the lowest standard curve point or LLOQ. The background noises or interferences responses at the retention time of the I.S. shall be acceptable if it is less than 5% of the mean response of the working I.S. Sensitivity was established from the background noise or response from six lots of blank plasma spiked with LLOQ concentration of analytes. The acceptable limits/error of accuracy and precision between the six replicates is ± 20%.

### Recovery

The recovery of nicotine and cotinine from matrix components (both plasma and brain) was estimated by the following equation:

$$Recovery = \frac{{Peak\,area_{test} }}{{Peak\,area_{reference} }} \times 100\%$$

Test refers to addition of known quantity of analyte spiked to the matrix before processing whereas reference solutions were prepared by spiking the same amount of analytes after processing the matrix blank. Five repeats of 3 concentrations (same as LQC, MQC and HQC) have been selected for recovery estimation. The recovery of IS was tested at the identical concentration used in the assay.

### Matrix effect

Since sample preparation involved simple protein crash using acetonitrile, matrix effect was investigated to ensure that precision, selectivity and sensitivity are not compromised by the matrix. Matrix effect was quantitatively evaluated at 3 concentrations (same as LQC, MQC and HQC) with five replicates each. Compared the post extraction spiked samples (test) with neat samples (reference) prepared in similar way without plasma or brain homogenate matrix. The acceptable limits of accuracy were set to 80–120% of nominal concentrations. Similarly, a precision ≤ 20% is considered acceptable.

Matrix effect of nicotine and cotinine from matrix components (both plasma and brain) was estimated by the following equation:

$$Accuracy = \frac{{Peak\,area_{test} }}{{Peak\,area_{reference} }} \times 100\%$$

### Stability

Stock solution stability is evaluated at 3 QC levels (LQC, MQC and HQC: n = 5) for 10 days at refrigerator temperature (4 °C). Stability of the stored samples will be compared against freshly prepared QC samples. Autosampler and bench-top stability tests were performed at 3 QC levels (LQC, MQC and HQC: n = 5). For autosampler stability, samples were prepared and kept in autosampler (4 °C) for 12 h, whereas another set of samples were left at ambient room temperature for 8 h prior to sample preparation. The concentrations were calculated against a calibration curve constructed from freshly prepared samples. Stability samples not exceeding the limit of accuracy (i.e., ± 15%) and precision (i.e., R.S.D 15%) are considered to be acceptable.

### Application of the method

#### Preclinical smoking model

**Fig. 2:**
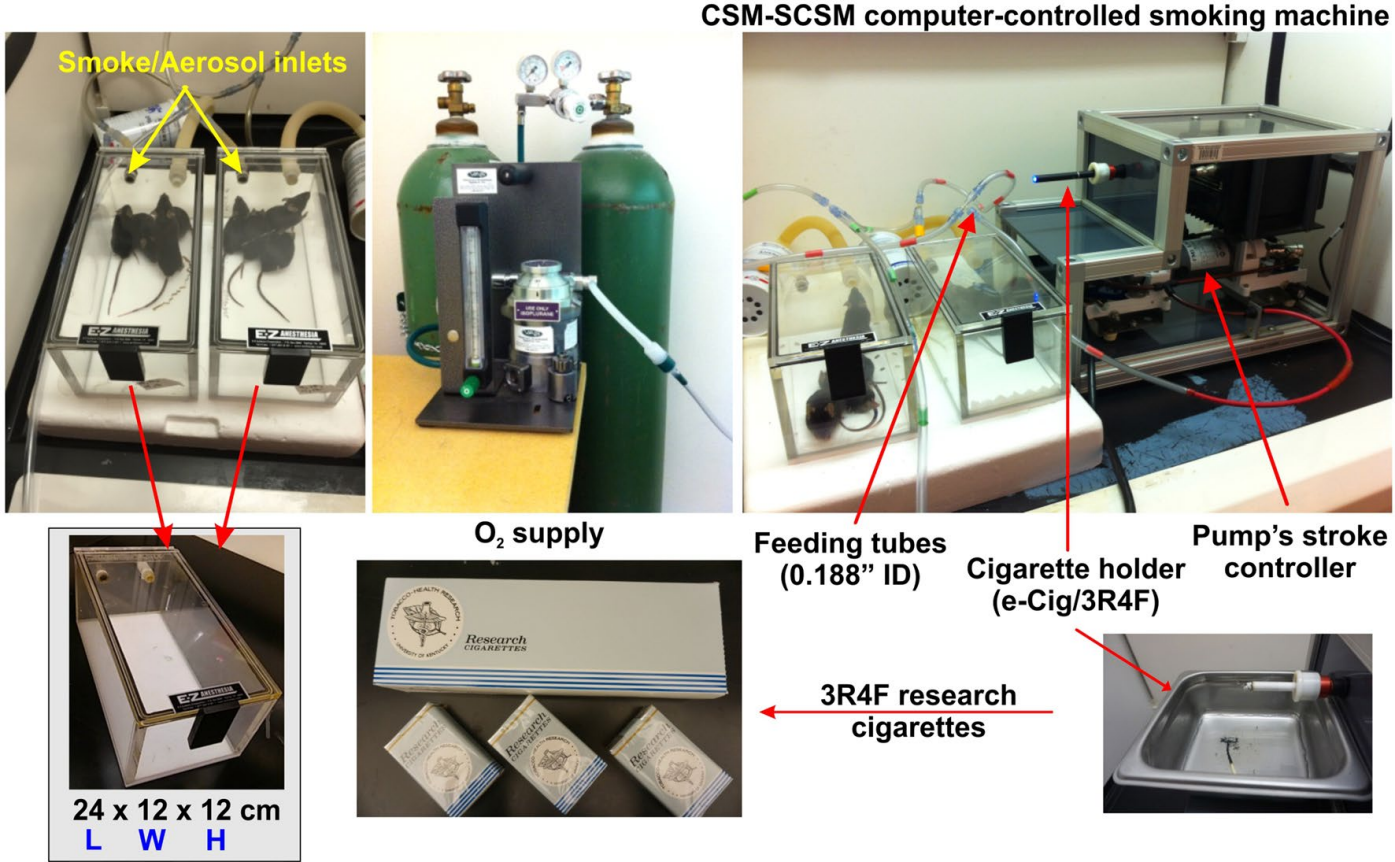
Preclinical smoking model in mouse. 8 (4 + 4) C57BL/6J mice are exposed (via direct inhalation) to side stream smoke generated from 3R4F research cigarettes (also compatible to automatic e-cigarettes). Tobacco smoke (TS) was generated and forced directly into two airtight smoking chambers (Dimension—24L × 12 W × 12H) housing the mice (4 mice/cage) by single channel cigarette smoking machine. The smoking inlet is dually connected to a feeding tube (0.188 in. ID; length-smoking machine to cage-30 cm, Oxygen cylinder to cage-150 cm) and a ventilator systems supplying O_2_ (2 L/min) at atmospheric levels (1 bar). During the interval between puffs, animals receive uninterrupted supply of normal oxygenated air

The animal studies were carried out in accordance with federal and state guidelines and the animal protocol for this work was approved by the Institutional Animal Care and Use Committee, TTUHSC, Lubbock, Texas. Total sixteen (two groups of eight) animals (C57BL/6J mice, Jackson Laboratories; male population; 8–10 weeks old; body weight: 20–25 g) were exposed (via direct inhalation) to side stream smoke from 3R4F research cigarettes (9.4 mg tar and 0.726 mg nicotine/cigarette—equivalent to full flavor brands; University of Kentucky) 6 times a day, 2 cigarettes/hour/8 animals every day for 7 and 14 days to simulate chronic human smoking behavior and achieve realistic plasma nicotine level of a chronic smoker (see Fig. 2). Tobacco smoke (TS) was generated and forced directly into two airtight smoking chambers (Dimension—24L × 12W × 12H) housing the mice (4 mice/cage) by CSM-SCSM cigarette smoking machine (CH Technologies, Westwood, NJ). TS was generated according to the modified Federal Trade Commission (FTC) standard protocol (1 puff/min, 35 mL puff depth volume, 4 s puff duration and 8 puffs in one session). The smoking inlet is dually connected to a feeding tube (0.188 inch ID; length-smoking machine to cage-30 cm, Oxygen cylinder to cage-150 cm) and a ventilator systems supplying O_2_ (2 L/min) at atmospheric levels (1 bar). During the interval between puffs, animals will receive uninterrupted supply of normal oxygenated air. Animals were transferred immediately from the smoking chamber to their regular housings with food and water supply once the smoking session is completed. Once the smoking cycle in last day was complete, 100 µL blood sample was collected immediately within 30 min by cardiac puncture, centrifuged at 1300 g for 10 min to obtain the plasma which was stored at − 80 °C. Following decapitation, brain was isolated and preserved at − 80 °C, homogenized in water (1:10 ratio) immediately before use.

Data were expressed as mean ± SEM and analyzed by one-way ANOVA using GraphPad Prism 6 Software Inc. (La Jolla, CA, USA). Post hoc multiple comparison tests were performed with Tukey’s or Dunnett’s test. *p* values < 0.05 were considered statistically significant.

## Results

### Calibration curve

**Fig. 3:**
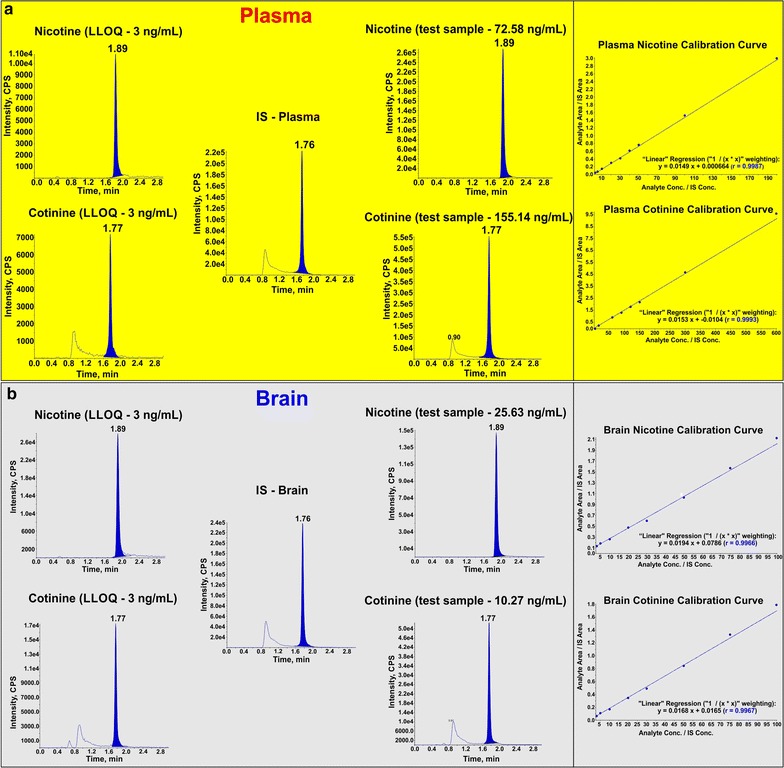
Representative peaks and calibration curves. Representative peaks of analytes (QC sample) at LLOQ (3 ng/mL), IS (20 ng/mL) and test samples in plasma (**a**) and brain (**b**) matrix. Note also the calibration curves for nicotine-plasma (3–200 ng/mL), cotinine-plasma (3–600 ng/mL), nicotine-brain (3–100 ng/mL) and cotinine (3–100 ng/mL) with r > 0.99

The calibration curve was consistently reproducible over the standard concentration range in both plasma and brain matrix (see Fig. 3). Calibration curve acquired by plotting peak ratio of analyte to IS versus nominal concentration using 1/X^2^ weighing factor gave regression > 0.99 in every occasion. The mean accuracy of the back calculated concentrations was found to be 99.98% with R.S.D 6.42% for nicotine and 100% with R.S.D 4.53% for cotinine in plasma and 99.99% with R.S.D 7.01% for nicotine and 99.99% with R.S.D 5.87% for cotinine in brain.

### Intra and inter-day accuracy and precision

Intra and inter-day accuracy and precision were measured by analyzing five replicates of QC samples at four different concentrations as shown in Table 2. For plasma the accuracy ranged from 107.06 to 84.34% with R.S.D ≤ 14.40% while for brain from 96.66 to 114.4% with R.S.D ≤ 14.48%.

**Table 2:** Intra and inter-run accuracy and precision of the method (n = 5)

Concentration (ng/mL)		Intra-run		Inter-run	
		Accuracy	R.S.D. (%)	Accuracy	R.S.D. (%)
Plasma					
Nicotine					
LLOQ	3	101.49	7.50	107.06	9.53
LQC	9	93.36	5.02	98.92	9.33
MQC	75	89.67	3.55	89.35	4.64
HQC	150	92.48	5.90	98.61	11.30
Cotinine					
LLOQ	3	84.34	3.7	94.29	10.99
LQC	9	105.98	6.93	100.25	14.40
MQC	225	90.55	1.24	89.96	6.97
HQC	450	97.89	9.5	98.31	8.13
Brain					
Nicotine					
LLOQ	3	104.26	4.36	102.59	14.48
LQC	9	111.01	4.93	107.28	5.65
MQC	40	114.45	7.36	109.44	9.10
HQC	80	113.46	6.91	110.70	7.51
Cotinine					
LLOQ	3	96.66	8.59	98.55	8.01
LQC	9	107.36	6.56	106.23	5.46
MQC	40	106.64	9.68	109.61	8.20
HQC	80	112.52	1.26	113.21	1.49

### Recovery and matrix effects

The recovery of both analytes and IS were estimated from the ratio of peak area for analyte spiked prior to matrix processing to that of analyte spiked following matrix processing. Recovery was measured in three different QC concentrations (LQC, MQC and HQC) from five replicates as mentioned in Table 3. Recovery of nicotine ranged from 95.05 ± 4.37% to 103.1 ± 2.69% in plasma and 94.96 ± 3.51 to 107.57 ± 6.46% in brain matrix whereas recovery of cotinine ranged from 89.22 ± 4.52 to 103.42 ± 4.13% in plasma and 90.37 ± 3.15% to 105.60 ± 3.71% in brain. Table 4 demonstrates the matrix effects of both plasma and brain. All concentrations met the acceptable limit except few of them marginally showed matrix effects.

**Table 3:** (%) Recovery (Mean ± SD) of analytes and IS (n = 5)

Concentration (ng/mL)		Recovery (%)	
		Analyte	IS
Plasma			
Nicotine			
LQC	9	95.05 ± 4.37	98.20 ± 1.86
MQC	75	103.10 ± 2.69	100.10 ± 2.36
HQC	150	95.00 ± 8.69	100.53 ± 1.30
Cotinine			
LQC	9	89.22 ± 4.52	98.20 ± 1.86
MQC	225	103.42 ± 4.13	100.10 ± 2.36
HQC	450	93.43 ± 2.49	100.53 ± 1.30
Brain			
Nicotine			
LQC	9	101.58 ± 1.00	95.79 ± 2.42
MQC	40	94.96 ± 3.51	95.14 ± 1.03
HQC	80	107.57 ± 6.46	95.57 ± 1.73
Cotinine			
LQC	9	99.01 ± 5.34	95.79 ± 2.42
MQC	40	90.37 ± 3.15	95.14 ± 1.03
HQC	80	105.60 ± 3.71	95.57 ± 1.73

**Table 4:** Matrix effects (mean ± SD) of plasma and brain on analytes (n = 5)

Concentration (ng/mL)		Matrix factor (%)
Plasma		
Nicotine		
LQC	9	80.32 ± 2.46
MQC	75	75.96 ± 18.97
HQC	150	77.37 ± 10.84
Cotinine		
LQC	9	90.00 ± 0.30
MQC	225	78.47 ± 4.13
HQC	450	87.08 ± 11.61
Brain		
Nicotine		
LQC	9	92.61 ± 5.12
MQC	40	91.37 ± 9.13
HQC	80	90.54 ± 7.29
Cotinine		
LQC	9	86.64 ± 2.38
MQC	40	89.65 ± 8.49
HQC	80	91.92 ± 5.16

### Stability

**Fig. 4:**
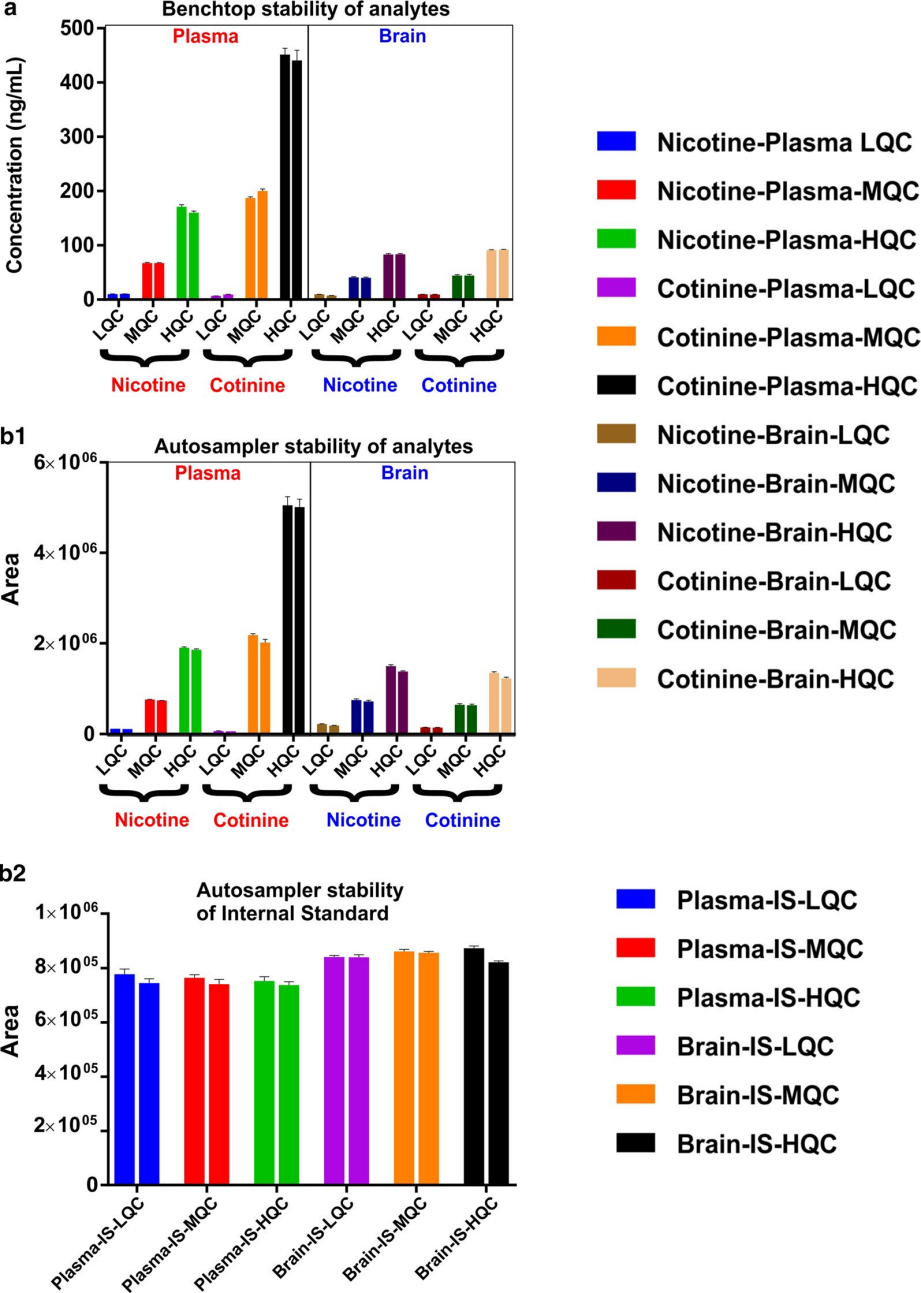
Benchtop and auto-sampler stability assay. Benchtop stability-Adjacent/grouped/paired columns (two columns with identical color) represent concentrations of control/non-stressed sample (measured immediately after sample preparation, first column) vs concentrations of stability/stressed sample (samples left at ambient temperature for 10 h prior to analysis-benchtop delayed, second column). No statistical difference in concentrations (between two adjacent columns) displays benchtop stability at LQC, MQC and HQC concentrations in both plasma and brain (**a**). *Autosampler stability*—Adjacent/grouped/paired columns (two columns with identical color) represent absolute peak area of control/non-stressed sample (measured immediately after sample preparation, first column) vs absolute peak area of stability/stressed sample (samples left at autosampler for 12 h prior to analysis, second column). No statistical difference in absolute peak areas (between two adjacent columns) shows autosampler stability of analytes- nicotine and cotinine (**b1**) and IS (**b2**) at LQC, MQC and HQC in both plasma and brain

Figure 4 depicting no significant change in concentrations/absolute peak area of analytes after delay in ambient temperature or in autosampler for 10 and 12 h respectively.

### Validating preclinical smoking model

**Fig. 5:**
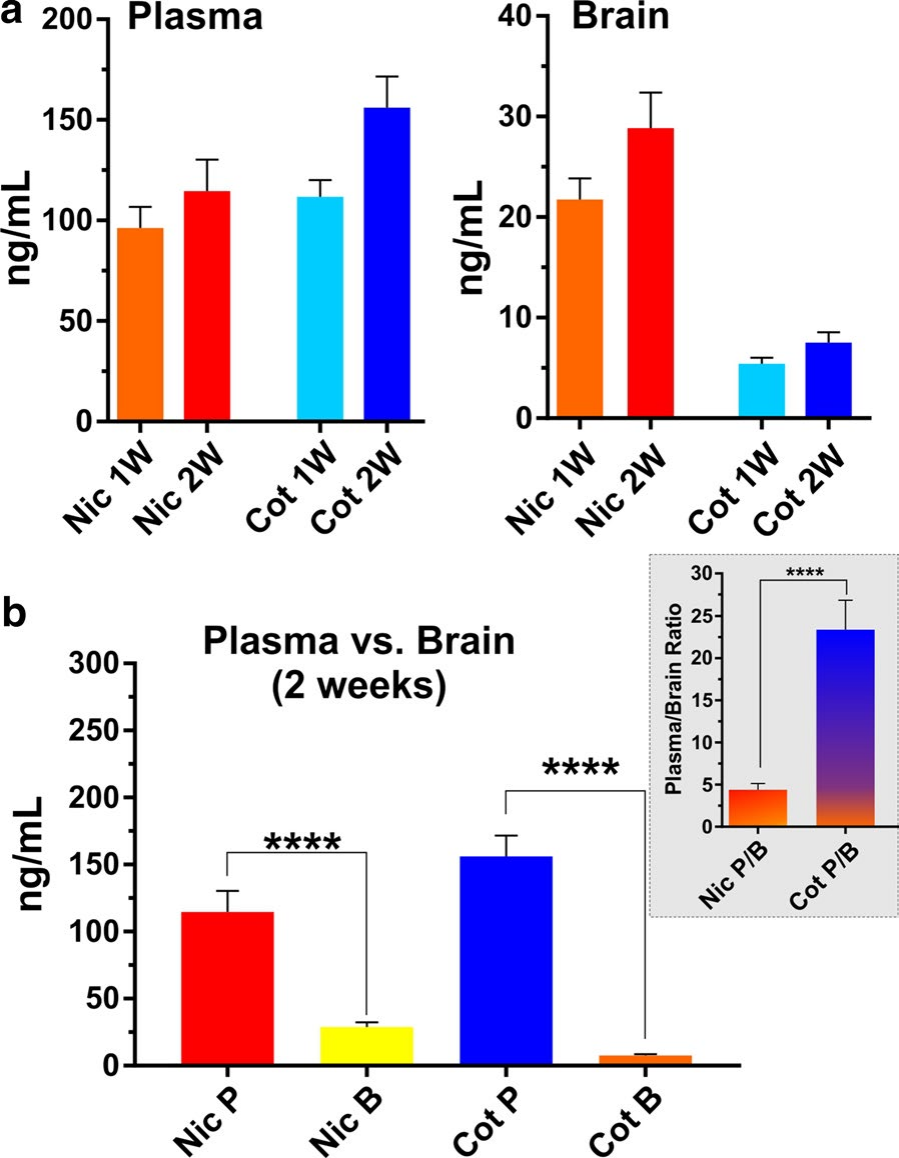
Plasma and brain levels of nicotine and cotinine in mouse. Nicotine and cotinine concentrations in mouse plasma and brain one and 2 weeks post exposure to TS (**a**). Plasma versus brain level and plasma : brain ratio of nicotine and cotinine 2 weeks post exposure to TS. **** *p* < 0.0001 plasma concentration of analytes against corresponding brain level, plasma:brain ratio of nicotine against plasma:brain ratio of cotinine (**b**)

Figure 5 shows the plasma and brain levels of nicotine and cotinine in mouse (n = 8 in each case) following one and 2 weeks of chronic exposure. After 1 week exposure, the nicotine concentration was found to be ~ 80 ng/mL while the cotinine concentration was ~ 112 ng/mL in plasma. Two weeks exposure produced a significant elevation of plasma cotinine levels compared to nicotine (> 150 ng/mL) as shown in Fig. 5. The concentrations of both nicotine and cotinine were significantly lower in brain than plasma. In brain, cotinine levels was nominal in comparison to nicotine following both one and 2 weeks of exposure. The plasma to brain ratio of nicotine was only five times but for cotinine it was approximately twenty-five times following 2 weeks of exposure.

## Discussion

WHO Report on the Global Tobacco Epidemic, 2013 reveals that tobacco smoking (TS) is accountable for approximately 6 million deaths per year globally with a projection of 8 million deaths annually by 2030. From a neuro-vascular perspective, TS has been associated with vascular endothelial dysfunction [25–27] in a causative and dose dependent manner [28]  primarily related to the TS content of reactive oxygen species (ROS) [27, 29], nicotine [30–35], and oxidative stress (OS)—driven inflammation [36]. As such TS is also a major prodromal factor for numerous central nervous system (CNS) disorders including Alzheimer’s, depression, cognitive impairment, stroke, and vascular dementia. Despite the strong evidence for an association between smoking and vascular impairment, the impact of TS exposure on the neuro-vascular unit (NVU) and its underlying pathophysiology has only been marginally addressed, thus leaving this a substantial understudied area.

Several preclinical models for exposing animals to TS were reported to understand the pathophysiology of the diseases caused by TS or TS derived nicotine. However, these models lack in simulating realistic human smoking pattern. Therefore, we report, a newly developed preclinical smoking model using C57BL/6 mice which novelty lies on a more realistic human-like smoking exposure pattern. This typically consists of smoking throughout the awake period with intervals in between cigarettes. Previously, reported exposure regimens do not provide such intervals resulting in continuous exposure ranging from 1 up to 5 h [37–39].

By contrast, our exposure protocol includes intervals in between exposure to cigarette smoke (puffs) during which animals receive uninterrupted supply of normal oxygenated air. In addition to these inter-puffs intervals, after 2 cigarettes/session are fully consumed, animals are transferred immediately from the smoking chamber back to their regular housings with food and water supply breathing normal oxygenated air before being subjected to the next smoking session. This cycle repeats 6 times per day (7 days a week) with a resting interval of 44 min in between sessions. The overall smoking exposure level is comparable to that of a heavy chronic smoker. Furthermore, animals undergoing smoking exposure are housed in a smoking chamber where animals are not restrained and/or under anesthesia. The smoking chamber can also house a larger number of animals (16 mice) in contrast to complex designed chambers [40] accommodating relatively less animals. Our exposure regimen is also feasible for both acute and chronic in vivo studies. Also, an additional advantage of our method, is that it can be adapted to routine plasma nicotine and cotinine level monitoring procedure (non-invasive) since analysis requires only 20 µL of blood which can easily be collected through the tail vein. Obviously, there are still unavoidable limitation to this model which is due to the fact that through human smoking behavior patterns are dynamic and tend to change during the course of smoking a cigarette. For example, smokers tend to take deeper and longer puffs at the beginning and as the cigarette get consumed these get shorter and smaller in volume. Inter-puff intervals instead, tend to be shortest at the beginning of the cigarette and longest near the end. These variable smoking patterns contrast with the static ones that can be currently reproduced by a smoking machine.

However, despite these limitation we believe that our model represents a significant step forward in the field and would be of significant help to researchers working to unmask the effects of TS/nicotine on CNS and its underlying pathophysiology. Findings from these studies can be positively correlated to human’s pathophysiology of TS toxicity and to further understand its negative impacts on a more detailed level. Further, in the past decade a number of alternative vaping products have hit the market, rapidly gaining consumers among adults and, especially, adolescents. Electronic nicotine delivery systems or e-cigarettes (e-Cigs) have become the sought-after product partly due to the belief that they are much safer than traditional cigarettes. Preclinical studies have shown that nicotine (the principal ingredient of e-liquid) can cause OS, exacerbation of cerebral ischemia and secondary brain injury [11, 34, 41]. Likewise, chronic e-Cig vaping could be prodromal to cerebrovascular impairment and promote cerebrovascular conditions similar to that associated with chronic TS. From this point of view, the health impact of e-Cig vaping is currently unknown and the limited research and dearth of regulatory for e-Cigs has become a critical public and regulatory concern. Thus, the development of a reliable in vivo model of chronic TS (and e-Cigs) exposure is even more crucial.

Currently available literature methods lack simpler sample preparation, sensitive method with small plasma volume and short run time. To validate the mouse model, we required a bioanalytical method for the simultaneous estimation of NT and CN both with 3 ng/mL as LLOQ in plasma and brain homogenate. For the first time we reported a method validation for the quantitative estimation of NT and CN using mouse plasma. The advantage of this method includes small sample volume (20 µL), simple protein crashing, sensitive method and short runtime of 3 min. This method can be further extrapolated to various studies involving different strains of mouse.

As mentioned earlier, the plasma level of nicotine of a chronic heavy smoker is up to 100 ng/mL whereas that of cotinine is ~ 250 to 350 ng/mL. Apart from interpersonal/genetic variation in metabolic enzymes responsible for nicotine metabolism and clearance, the plasma concentration of nicotine varies widely depending the interval between sample collection and last time smoked. We collected mouse plasma samples within less than 30 min following last cigarette smoked. Many reports suggest higher cotinine:nicotine plasma ratio as we observed here in mouse model, fast sample collection explains the fact that, plasma nicotine was not exposed to CYP450 s for enough period of time to be metabolized and cleared off.

There are controversial reports regarding brain uptake of cotinine. Some reports suggest, cotinine is polar in nature compared to nicotine and it does not cross BBB, the presence of cotinine in brain is due to presence of metabolic enzymes in brain that converts nicotine penetrated in brain into cotinine while other reports demonstrated brain uptake of cotinine itself [6]. We here show that, the cotinine concentration in brain is significantly less compared to plasma and brain nicotine level.

## Conclusion

A simple, rapid, sensitive and specific UHPLC-MS/MS method has been developed and validated for quantification of NT and CN in mouse plasma and brain homogenate as per the regulatory guidelines. The method showed suitability for validating preclinical animal smoking/vaping model using C57BL/6J mice. The simple protein crash method using 8 volumes of acetonitrile furnished consistent and reproducible recoveries for NT, CN and IS from plasma as well as brain homogenate. The simplicity of the extraction process and use of very less sample volume, and sample turnover rate of 3 min per sample, make it an attractive procedure in high-throughput bioanalysis of NT and CN. From the results of all the validation parameters, we can conclude that the developed method can be useful for any further studies in mice where blood volumes are limited per time point. A method with small sample volume from animals involved in research is in line with 3R principle of replace, reduce and refine animals in research.

## Abbreviations

CNS: central nervous system
CN: cotinine
e-Cigs: e-cigarettes
GC–MS: gas chromatography-mass spectrometry
HQC: high quality control
IS: internal standard
LC–MS/MS: liquid chromatography-tandem mass spectrometry
LQC: low quality control
LLOQ: lower limit of quantification
MQC: middle quality control
MRM: multiple reaction-monitoring
NVU: neuro-vascular unit
NT: nicotine
OS: oxidative stress
QC: quality control
ROS: reactive oxygen species
TS: tobacco smoking
